# Supplementary material for: Analysis of virulence potential of Escherichia coli O145 isolated from cattle feces and hide samples based on whole genome sequencing
Source: PLoS One. 2019 Nov 27;14(11):e0225057. doi: 10.1371/journal.pone.0225057 (PMC6881001; doi:10.1371/journal.pone.0225057)
Supplement: Attachment — Submitted filename: RESPONSE TO REVIEWERS-OCT 2 2019.docx [file pone.0225057.s001.docx]

**Author’s Response to Reviewers**

**Reviewer #1**

1. No affiliation for number 3 (Lance W. Noll3)

***Author’s Response:*** *Affiliation has been added. Please see line 14*

2. Abstract and elsewhere in the manuscript: the “average genome size” should be changed to say the “approximate average genome size” as these are just draft genomes.

***Author’s Response:*** *We agree that average genome size is somewhat misleading. However, approximate may not be the right word either. We have chosen to use average draft genome, which is an accurate description.*

3. Introduction, lines 58 and 63. Remove “have” from the sentence.

***Author’s Response:*** *Corrected. Please see lines 57 and 62*

4. Materials and Methods, lines 82-85. I was surprise to see such few isolates used to make a major claim of cattle STEC O145:H28 isolates potential can cause human illness. There were only two human isolates used in this study. I don’t know how there can be any power to your statistical analysis with just two strains. I am not even sure the 16 cattle feces isolates would be enough to represent all the genetic diversity of STEC O145:H28 in cattle. There are sequenced STEC O145 that can be downloaded from Genbank to increase the number of human isolates and increase the diversity of the isolates used in this study.

***Author’s Response:*** *We agree that 16 isolates from feces is a small number and is not robust enough to describe genetic diversity. However, 53 isolates from hide is a decent number compared to what is publically available. In all our fecal prevalence studies in feedlot cattle, we obtained a number of O145 strains, but only a few were positive for Shiga toxin gene. At NCBI, as of today, whole genome sequences of 364 strains are available, which include draft and complete genomes, and sequence reads, excluding the strains that we have deposited. Of the 364 strains, a majority were human clinical strains (n=258) and only 16 strains were of cattle origin. Of the 16, 5 were from feces, 1 from hide, and for the remaining 10, source was not identified. Therefore, our study provides sequence analyses of a substantial number of strains from cattle, particularly strains from hides. At the same time, we are not sure adding more human strain sequences into our analyses would be beneficial and is likely to dilute the main objective of the study, which was to analyze cattle strains. Please see lines 316-320*

5. Materials and Methods, lines 82-85. The two references sited (14 and 15) are both from cattle feces. I didn’t see where any of them were from an abattoir. How did you select which cattle fecal isolates to sequence? Between the two papers in the reference, there should have been well over 100 isolates you could sequence. Were the hide samples from the abattoir from the same or feed yard? They are closely related which makes me think they are epidemiologically related. How do you explain the clustering of cattle feces and hide isolates on the tree when the hide isolate more than likely came from feces?

***Author’s Response:*** *We have corrected this. The fecal strains used in this study were from ref #14 and 16. The hide strains were from a different study, which we have cited now (See ref #;16 Please see line 84-87) Hide samples were collected from an abattoir as part of a different study (they were not collected from the same animal/feedlot from which the fecal samples were collected) and we were surprised to find high number of O145 strains positive for Shiga toxin gene. The only explanation for high prevalence on hide is that the hide strains were from different feedlots and time points. It is generally accepted that the hide strains originate from fecal contamination. The reason why the fecal and hide strains clustered separately in our study is likely because they were from different feedlots.*

6. Discussion, line 298. Remove “the” before ”fecal strains …”.

***Author’s Response:*** *Complied. Please see line 310*

**Reviewer #2**

1. I suggest using “big six non-O157 STEC” instead of “top-7 STEC”.

***Author’s Response:*** *Complied. Please see line 23*

2. I suggest mention that your sample size is small (in the conclusion of abstract and discussion)

***Author’s Response:*** *Agreed. Please see lines 43-45, 316-320*

3. Lines 45 and 46 are not suitable for initiating of introduction, I suggest removing them.

***Author’s Response:*** *Removed*

4. Line 69: Please mention the time of sampling for O145 collection? How many years? How many months? Since what year and month to what year and month?

***Author’s Response:*** *Complied. Please see lines 84-89*

5. Line 86 data is more appropriate for results section not for materials and methods.

***Author’s Response:*** *We included the prevalence of the three major virulence genes here only because they were based on PCR assays previously published.*

6. Line 99: Correct the “))”

***Author’s Response:*** *Corrected*

7. Line 144: "[260 (224-291)]" seems to be better than "(260 [224-291])". Check it.

Author’s Response: Complied

8. Because of the small sample size, I disagree with percentage in the text and table 2. Consult with an epidemiologist.

***Author’s Response:*** *We included percentages to make it easier to compare. We have removed the percentages.*

9. in figure captions: What algorithm is used? (For example: UPGMA), what similarity coefficient is used? (For example: Dice)

***Author’s Response:*** *We have included the description in the Figure legend (Please see lines 228-232)*

10. In discussion, you can compare your stx subtypes with some other studies to show the importance of cattle as a disseminator of the subtypes:

***Author’s Response:*** *Complied. Please see lines 248-261*
